# Research trends and hotspots regarding treatment of obstructive sleep apnea

**DOI:** 10.3389/fneur.2023.1268639

**Published:** 2023-10-18

**Authors:** Xia Yang, Yi Wen, Shiqi Xie, Jinglan Chen, Yue Liu, Jianrong Zhou

**Affiliations:** College of Nursing, Chongqing Medical University, Chongqing, China

**Keywords:** obstructive sleep apnea, OSA, therapy, bibliometric analysis, hotspots

## Abstract

**Background:**

Obstructive sleep apnea (OSA) is a type of sleep-disordered breathing disease, with high prevalence and multiple complications. It seriously affects patients’ quality of life and even threatens their lives. Early and effective treatment can significantly improve patients’ health conditions.

**Objective:**

In this study, the main treatment methods, research hotspots and trends of OSA were summarized through bibliometric and visualization analysis.

**Methods:**

From the Web of Science Core Collection database, articles on the treatment of OSA from 1999 to 2022 were obtained. CiteSpace and VOSviewer were comprehensively used to visualization of journals, co-authorship of countries, institutions and authors, co-citation of references, keywords cluster and burst.

**Results:**

A total of 2,874 publications were obtained, of which 2,584 were concerned adults and 290 about children. In adults’ research, *Sleep and Breathing* is the most published journal (280, 10.84%), the largest number of publications come from the United States (636,24.61%) and the University of Sydney (88, 3.41%), and Pepin JL is the most published author (48, 18.58%). In children’s studies, *International Journal of Pediatric Otorhinolaryngology* is the most published journal (41, 14.14%), the maximum number of publications were also from the United States (123, 42.41%), with the University of Pennsylvania (20, 6.90%) and Marcus CL (15, 5.17%) being the most published institutions and authors. High-frequency keywords for adults’ researches include positive airway pressure, oral appliance, surgery and positional therapy. On these basis, children’s studies also focus on myofunctional therapy, rapid maxillary expansion and hypoglossal nerve Stimulation.

**Conclusion:**

Over the past two decades, research in the field of OSA therapeutics has experienced significant growth in depth and breadth. The author cooperation network has already established a solid foundation, while there is potential for further strengthening the cooperation network between countries and institutions. Currently, positive airway pressure and surgery are the primary treatments for OSA in adults and children. Future research will focus on multidisciplinary combination targeted therapy, which presents a key area of interest and challenge.

## 1. Introduction

Obstructive sleep apnea (OSA) is a prevalent clinical syndrome characterized by repeated collapse and obstruction of the upper airway during sleep, resulting in sleep apnea, hypoventilation, intermittent hypoxia and hypercapnia (1, 2). Previous studies have reported a prevalence of 24% in adult males and 9% in females (3). And it affects approximately 1 to 3% of children (4). Global research on the prevalence and burden of OSA suggests that nearly 1 billion adults aged 30–69 years may suffer from OSA, with an estimated 425 million individuals experiencing moderate to severe disease that typically requires treatment. China leads with an estimated 176 million cases (5). Intermittent hypoxia is the primary pathophysiological feature of OSA, characterized by rapid reoxygenation following recurrent episodes of hypoxia at night. This process triggers the production of inflammatory factors (such as C-reactive protein, tumor necrosis factor α, interleukin-10) and oxidative stress (6–8), leading to complications in the heart, brain, lungs, and blood vessels (9–11). OSA has predictable negative economic consequences and is associated with an increased risk of car accidents (12). It also negatively impacts children, who often experience cardiovascular issues such as pulmonary hypertension, systemic hypertension, left ventricular hypertrophy, reduced oxygen saturation, changes in vascular tone (13), growth disorders (14), as well as neurobehavioral functioning, including poor academic performance and difficulties in social and emotional development (15–17).

Currently, a range of therapeutic approaches is employed for the treatment of OSA, including surgical and nonsurgical interventions. These approaches aim to eliminate upper airway obstruction, prevent collapse, and correct hypoxemia during sleep (18). OSA has long been recognized as a heterogeneous disease (19), with the pathogenesis in children being less well-defined, primarily attributed to adenotonsillar hypertrophy and mouth breathing (20). In adults, pathogenesis involves anatomically compromised upper airway stenosis or collapse, impaired control and function of the pharyngeal dilator muscles, a heightened likelihood of awakening during airway stenosis (low arousal threshold), and unstable respiratory control (high loop gain) (21, 22). Each phenotype represents a potential therapeutic target, and the relative contribution and choice of treatment may vary from patient to patient, as does patient adherence to treatment (23). Yet, in various study, the singular focus on the phenotypic alteration of impaired upper airway anatomy and the reliance on symptomatic treatment modalities may impede the long-term progress of therapeutic advancements in the field of OSA. Rather than relying on one-size-fits-all or experience-based approaches, identifying pathogenetic features of OSA and exploiting therapies that target specific phenotypes, may be possible to personalize the management of chronic health conditions and optimize patient prognosis. Therefore, we elucidated the development trajectory of research on OSA treatment through bibliometric analysis. We attempt to answer: What are the research priorities and directions at this stage? What are the emerging therapeutic approaches and possible treatment mechanisms? The study aim is to provide a basis for the future development of OSA treatment toward precision medicine.

Bibliometrics is the analysis of published information (e.g., books, journal articles, datasets, blogs) and its associated metadata (e.g., abstracts, keywords, citations) using statistical data to describe or show relationships between publications (24). Tools are crucial for scientific research, helping scientists to identify research questions, analyze data, visualize results and disseminate knowledge. CiteSpace and VOSviewer, the bibliometric tools utilized in this study, are widely employed in the field (25). CiteSpace is a Java-based information visualization software primarily relies on co-citation analysis theory and the pathFinder algorithm. It assesses the literature of a specific domain, elucidating the critical paths and inflection points in the evolution of a subject area over time. It allows for a comprehensive understanding of the field’s development process and trends through the timezone and timeline views (26). CiteSpace is highly applicable to a wide range of users, including scientists, researchers in science policy, and graduate students (27). VOSviewer employs a data standardization method based on probability theory. It offers various visualization views encompassing keywords, co-institutions, and co-authors. These views include visualization of network, overlay and density, providing users with easy charting capabilities and visually appealing images (28).

## 2. Methods

### 2.1. Data sources and search strategies

With over 12,000 prestigious journals, Web of Science (WoS) severs as a comprehensive database encompassing a broad range of academic literature, serving researchers worldwide across diverse fields of knowledge (29). Compared to databases like Scopus and PubMed, WoS holds unrivaled recognition for its exhaustive and reliable resources, making it the foremost choice for conducting meticulous bibliometric analyses (30, 31). All publications were searched from WoS Core Collection database, which includes the Science Citation Index Expanded, Current Chemical Reactions Expanded, and Index Chemicus. The retrieval time range was from 1999 to 2022. The search formula was TS = (“OSAHS” OR “OSA “OR “OSAS” OR “snoring” OR “obstructive sleep apnea hypopnea syndrome” OR “obstructive sleep apnea”) AND TS = (“therapeutic method” OR “therapy” OR “treatment” OR “remedy”), with the search type limited to “article.” All searches were performed on a single day (15 August 2022) to minimize bias resulting from daily updates to the database. A total of 11,914 documents were obtained. The content of the downloaded records was selected as “full record and cited references,” and each record in the downloaded file included the author, title, abstract, keywords and citations.

### 2.2. Inclusion and exclusion criteria

Original studies focusing on the treatment of OSA published between 1999 and 2022 were included. Exclusion criteria were as follows: redundant publications; book chapters, early access articles, conference papers, retracted publications, reviews, trend analyses and similar formats; articles without abstract or full text; non-English articles.

### 2.3. Literature screening and bibliometric analysis

**Figure 1:**
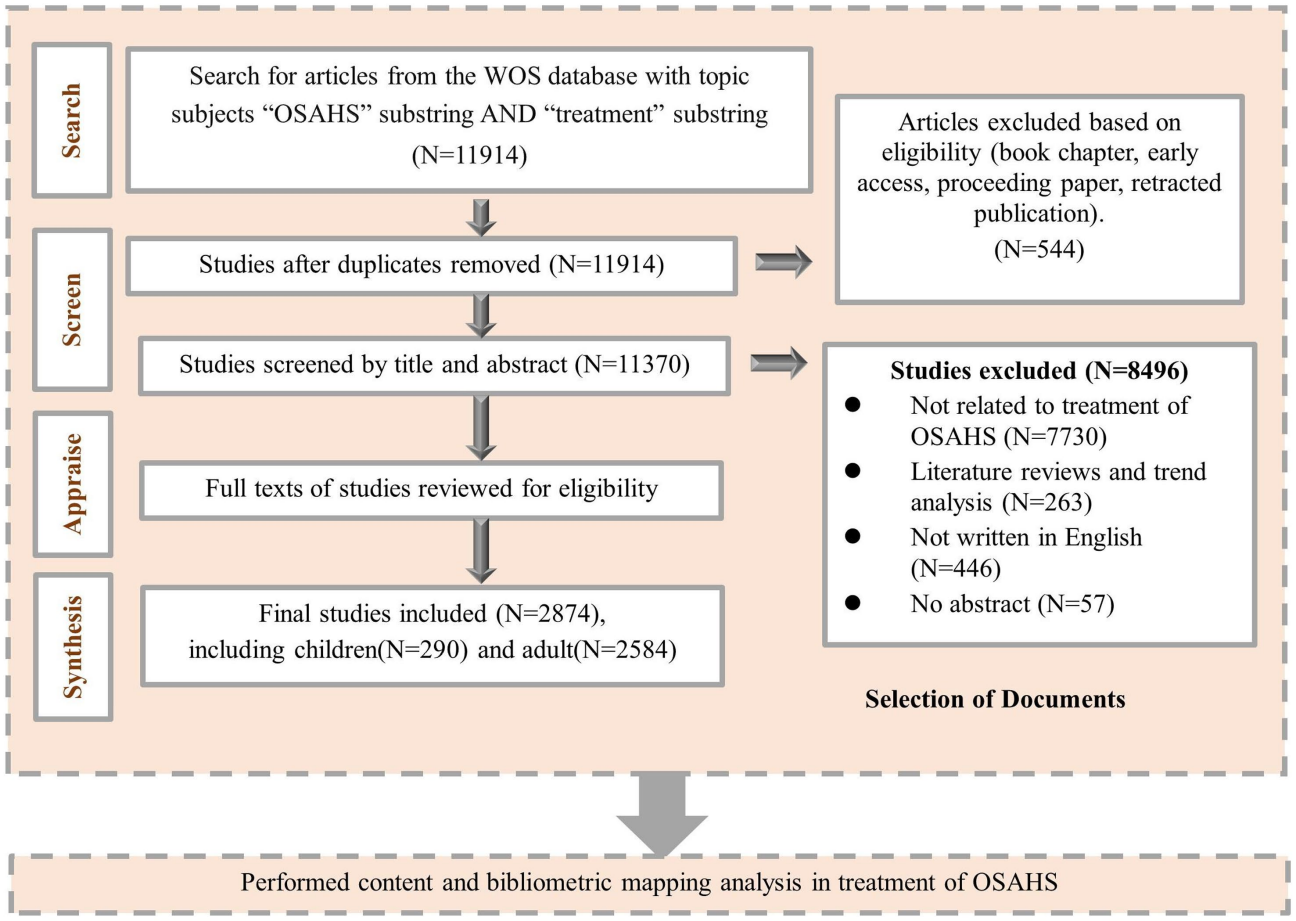
Article selection process.

CiteSpace 6.2.R2 was utilized to examine the search results and eliminate duplicated papers, book chapters, early access articles, proceeding papers, and retracted publications. The remaining screening process was performed independently by two researchers, involving the review of titles and abstracts based on the predetermined inclusion and exclusion criteria. In case of disagreement, an independent full text review or discussion with knowledgeable corresponding authors was necessary. Statistical Product and Service Solutions (SPSS) software (version 25.0, IBM Corporation, Armonk, NY, USA) was used to calculate Cohen’s Kappa coefficient to evaluate the Inter-rater reliability. Figure 1 depicted the precise literature screening procedure.

The bibliometric analysis and scientific mapping were performed using the following software systems: CiteSpace 6.2.R2, VOSviewer 1.6.19, and Microsoft Office Excel 2021. Based on the intrinsic features of WoS database, we described the fundamental characteristics of the publications, including publication type, quantity, year, country, institution, authors, per-part citation index, H-index, Impact Factor (IF) 2021, and Journal Citation Report (JCR) categories. The H-index signifies that an individual or entity has published H papers, each of which has garnered at least H citations from other publications (32). This metric is commonly employed to assess the academic productivity and impact of researchers, and it can also be applied to evaluate the publication output of countries, journals, and institutions (33). The impact factor (IF) of a journal is a metric used to assess its significance or ranking (34). It is calculated by determining the average number of citations that articles published by the journal received in a calendar year over the preceding 2 years (35). Additionally, quartile rankings are assigned based on the journal’s performance in the Journal Citation Indicator, as per the JCR 2021 standard.

Based on CiteSpace, we performed a network analysis of keywords, including keyword co-occurrence, clustering, timeline graphs and emergent words. The specific parameters employed were as follows: time range (1999–2022), years per slice (1 year), term sources (title, abstract, author keyword, keyword plus), node type (keyword), selection criteria (g-index: *k* = 25 or *k* = 20), pruning (pathfinder, pruning sliced networks), and the remaining settings were default. The timeline graph depicts the relationship between clusters and the temporal distribution of literature within a specific cluster. It uses the vertical coordinate to represent the cluster to which a node belongs and the horizontal coordinate to represent the publication time. Bursting words are terms that are frequently cited over a certain period and can indicate emerging or cutting-edge research topics (36). These keywords are indicative of the investigation of cutting-edge topics over time. For the analyses of adult and child studies, burst keyword analysis was conducted using Burmap, with γ values set to 1 and 0.5, respectively. The parameter for minimum durations was set to 1. Categorization was performed based on the start year, sorted in ascending order.

Based on VOSviewer, we conducted an analysis of collaborative relationships and reference co-citation relationships among countries, institutions, and authors. Furthermore, we visually represented the collaborative network overlay for core authors. The overlay view incorporates a time dimension, which intuitively reflects the scholarly activity of researchers in recent years through a gradient of colors. According to Price’s law, a core group of authors can be considered to have formed in the field if a stable core group of authors accounts for 50% of the total number of papers (37). The minimum number of publications by core authors in a specific field can be calculated using the following formula: $m=0.749\times \surd n_{\text{max}}$ . The $n_{\text{max}}$ in the formula indicates the number of publications in the field by the most prolific authors.

Each node in the map, generated by the two software mentioned above, corresponds to a country, organization, author, or keyword. The size of each node represents its frequency of occurrence, while the color of the nodes indicates their cluster or temporal distribution. The lines connecting the nodes represent the links (LS), and a thicker line indicates a tier link between the two nodes (28). The total link strength (TLS) and centrality metrics are used to assess the importance of nodes within a given network. Excel was used to produce line charts and trilinear tables.

## 3. Results

### 3.1. Characteristic of included papers

**Figure 2:**
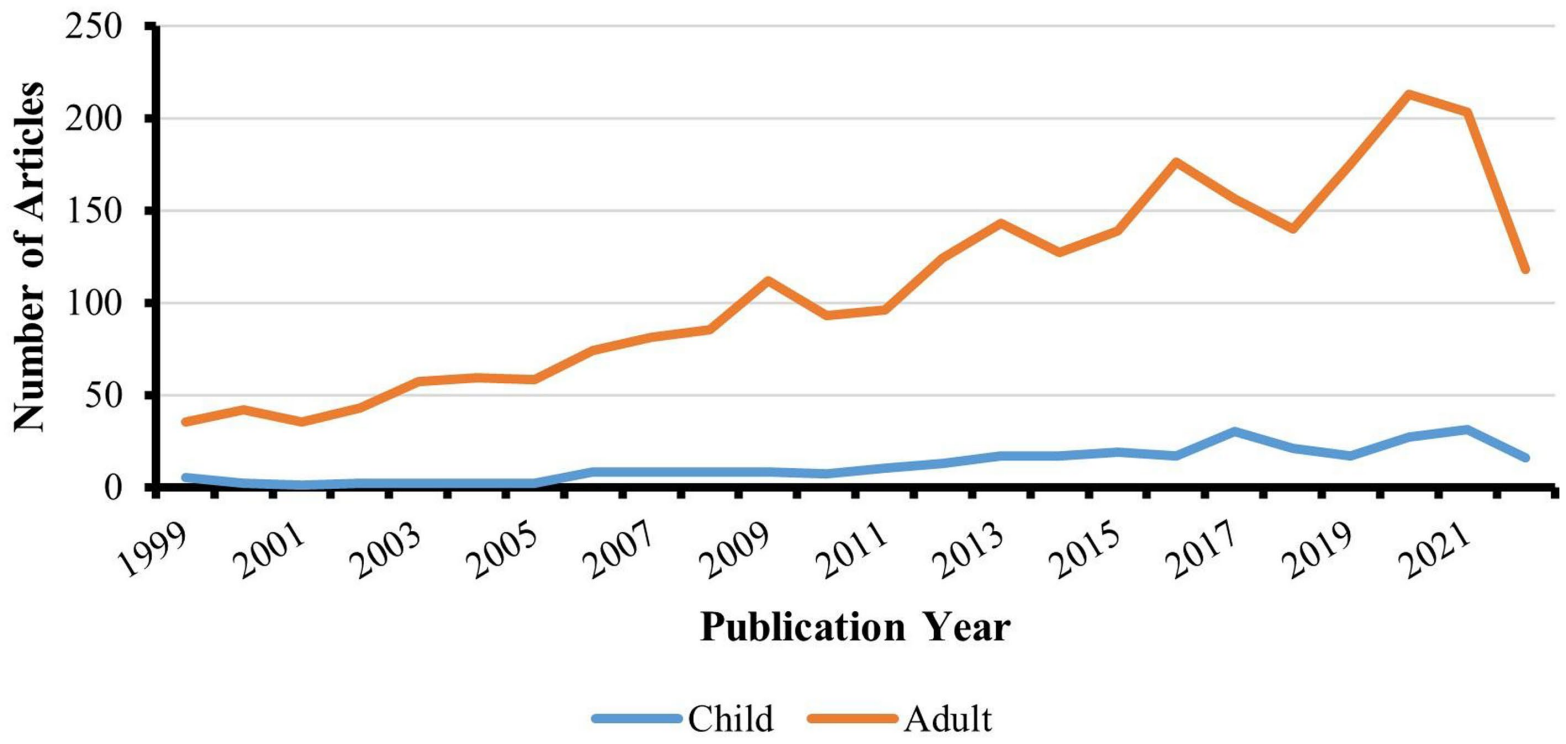
Publication trends over time.

The screening results of the two researchers showed a high level of agreement (kappa value 0.94). Ultimately, 2,874 valid articles were included, of which 290 were related to pediatric OSA, and 2,584 were related to adult OSA. The characteristics of the included papers were illustrated in Figure 2, displaying their distribution from 1999 to 2022. The number of publications has shown an overall rising trend. From 1999 to 2008 and from 2010 to 2011, the number of papers related to adults remained below 100. However, after 2008, the number of publications increased rapidly, reaching its peak in 2020 and 2021. On the other hand, the number of articles focusing on children reached its first level in 2017 and the second level in 2021, although none exceeded 50. These findings suggest that the treatment of OSA has gained attention as a research area worthy of concern and has gradually developed over the past two decades, while the focus on children is relatively weaker compared to adults.

### 3.2. Analysis of journals

We conducted an analysis of publication volume, average citation per publication, IF, and H-index for the journals. Table 1 presents the top 10 journals that have published the most articles on adult OSA treatment. These journals are predominantly focused on sleep respiration and otorhinolaryngology-head and neck surgery. The publication volume of these journals accounts for nearly 50% of all publications on adult OSA treatment, with *Sleep and Breathing* ranking first with 280 relevant articles. *Journal of Clinical Sleep Medicine* and *Sleep Medicine* secured the second and third positions with 192 and 127 publications, respectively. 70% of the top 10 journals have an H-index greater than 30. The *American Journal of Respiratory and Critical Care Medicine* holds the highest average citation per publication (136.54) and H-index (55), positioning it as an influential journal with an IF rating (30.528) second only to the *European Respiratory Journal* (33.795).

**Table 1:** Top 10 journals of OSA treatment in adults and children by publications.

Rank	Journal (Adults)	Publications (%)	Average Citation/Publication	IF(Q)*/*H* index	Journal (Children)	Publications (%)	Average Citation/Publication	IF(Q)*/*H* index
1	Sleep and Breathing	280(10.84)	15.81	2.655(3)/35	International Journal of Pediatric Otorhinolaryngology	41(14.14)	18.78	1.626(4)/18
2	Journal of Clinical Sleep Medicine	192(7.43)	23.13	4.324(2)/40	Journal of Clinical Sleep Medicine	24(8.28)	26.08	4.324(2)/14
3	Sleep Medicine	127(4.92)	26.02	4.842(2)/33	Sleep Medicine	21(7.24)	26.05	4.842(2)/11
4	Sleep	126(4.88)	63.26	6.313(2)/54	Otolaryngology Head and Neck Surgery	14(4.83)	14.07	5.591(2)/8
5	Chest	99(3.83)	75.19	10.262(1)/51	Laryngoscope	11(3.79)	40.36	2.970(2)/7
6	American Journal of Respiratory and Critical Care Medicine	70(2.71)	136.54	30.528(1)/55	Sleep	11(3.79)	39.09	6.313(2)/11
7	Laryngoscope	68(2.63)	30.84	2.970(2)/26	Sleep and Breathing	10(3.45)	28.2	2.655(3)/8
8	European Respiratory Journal	67(2.59)	61.91	33.795(1)/41	Jama Otolaryngology Head Neck Surgery	9(3.10)	16.22	8.961(1)/7
9	European Archives of Oto Rhino Laryngology	59(2.28)	12.00	3.236(3)/14	Pediatrics	9(3.10)	95.11	9.703(2)/9
10	Otolaryngology Head and Neck Surgery	55(2.13)	24.73	5.591(2)/22	Pediatric Pulmonology	8(2.76)	23.38	4.09(3)/6

*F and Q in category according to Journal Citation Reports (2021). IF, impact factor; Q, quartile.

Table 1 displays the top 10 journals with the highest publication output on children OSA treatment. These journals published 50.7% of the articles, with 70% of the journals having an IF above 3. Three of these periodicals are devoted to pediatric research, with the *International Journal of Pediatric Otorhinolaryngology* securing the first position with 41 relevant articles and an H-index of 18. Similar to adults, the *Journal of Clinical Sleep Medicine* and *Sleep Medicine* ranked second and third in terms of volume of publications, respectively. *Pediatrics* published nine articles, having the highest average citation per publication (95.11) and IF (9.703).

### 3.3. Analysis of countries, organizations, and authors

Co-authorship analysis was conducted to examine the relationship between countries, organizations, and authors based on the number of co-authored papers.

#### 3.3.1. Analysis of countries

**Figure 3:**
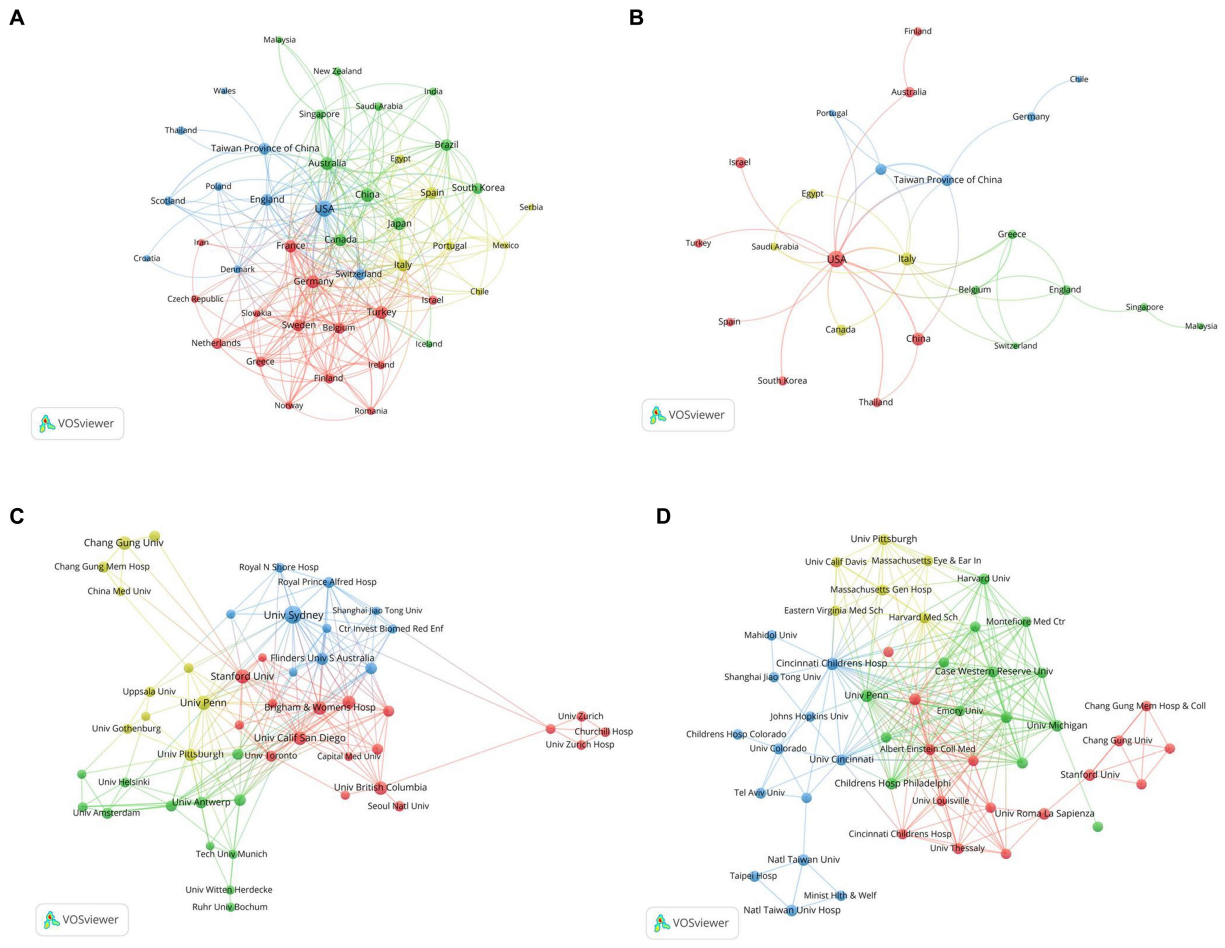
The cooperative countries and organizations in the field of OSA treatment. **(A)** Co-authorship network visualization of countries in adults. **(B)** Co-authorship network visualization of countries in children. **(C)** Co-authorship network visualization of organizations in adults. **(D)** Co-authorship network visualization of institutions in children. Each node in the network represents a country or organization. The size of the node indicates the frequency of its occurrence. The nodes are color-coded to represent the different clusters they belong to. The lines connecting the nodes represent links, with the thickness of the lines indicating the strength of the connections.

In this study, a co-authorship analysis was conducted for the 66 identified countries in adult OSA treatment publications. The minimum number of papers per country was set at 0, and the minimum number of citations was set at 5. Out of these countries, 45 met the threshold, and the results are presented in Figure 3A. Based on the publication count, the top 3 countries are the USA (Documents = 636, citations = 24,887, TLS = 365), China (Documents = 205, citations = 4,198, TLS = 65), and Australia (Documents = 188, citations = 9,945, TLS = 137). Additionally, there are 10 other countries with more than 100 publications, including Germany, Italy, Turkey, and France. Collaboration between countries is evident, with the USA and Australia showing the highest level of collaboration (LS = 45). The USA and Taiwan Province of China (LS = 31) and the USA and Germany (LS = 27) are followed. Interestingly, Lithuania (Documents = 5, citations = 11,579, TLS = 0) did not cooperate with any of the countries.

Analogously, a co-authorship analysis was conducted for the 37 identified countries in the included children’s publications. The minimum number of papers per country was set at 1, and the minimum number of citations was set at 0. All countries met the threshold, and the results are depicted in Figure 3B. In terms of publications, the top 3 countries are the USA (Documents = 123, citations = 4,994, TLS = 36), China (Documents = 32, citations = 398, TLS = 6), and Italy (Documents = 28, citations = 844, TLS = 15). Collaboratively, the USA and Taiwan Province of China demonstrated the highest level of collaboration (LS = 8), and collaboration between the USA and Italy (LS = 5) followed.

#### 3.3.2. Analysis of organizations

An organizational co-authorship analysis was conducted for the 2,985 identified organizations in the included adult publications. The minimum number of papers per organization was set at 16, and the minimum number of citations was set at 0. Out of these organizations, 50 met the threshold and were interconnected, as depicted in Figure 3C. The University of Sydney (Documents = 88, citations = 5,330, TLS = 115), the University of Pennsylvania (Documents = 52, citations = 2,595, TLS = 40), and Stanford University (Documents = 50, citations = 2,317, TLS = 36) emerged as the leading organizations. Among them, Brigham & Women’s Hospital and Harvard Medical School exhibited the closest collaboration (LS = 29). It is worth mentioning that only two organizations, Kyoto University and the University of Milan, did not collaborate with any other organization.

A co-authorship analysis was performed for the 393 identified organizations in the included children’s literature. The minimum number of papers per organization was set at 3, while the minimum number of citations was set at 0. Sixty-eight organizations met the threshold, as illustrated in Figure 3D. The University of Pennsylvania (Documents = 20, citations = 1,886, TLS = 83), Cincinnati Children’s Hospital Medical Center (Documents = 14, citations = 1,011, TLS = 70), University of Roma La Sapienza (Documents = 12, citations = 656, TLS = 5), Stanford University (Documents = 11, citations = 343, TLS = 13), University of Chicago (Documents = 11, citations = 1,421, TLS = 39), and National Taiwan University (Documents = 10, citations = 153, TLS = 19) were the six organizations with 10 or more publications. Notably, the University of Pennsylvania collaborates closely with Cincinnati Children’s Hospital Medical Center (LS = 9), and National Taiwan University collaborates with National Taiwan University Hospital, both demonstrating high levels of collaboration (LS = 9). Overall, it is evident that institutions in Europe and the Americas exhibit close collaboration in external research endeavors. However, physical distance still presents a constraint.

#### 3.3.3. Analysis of authors

**Figure 4:**
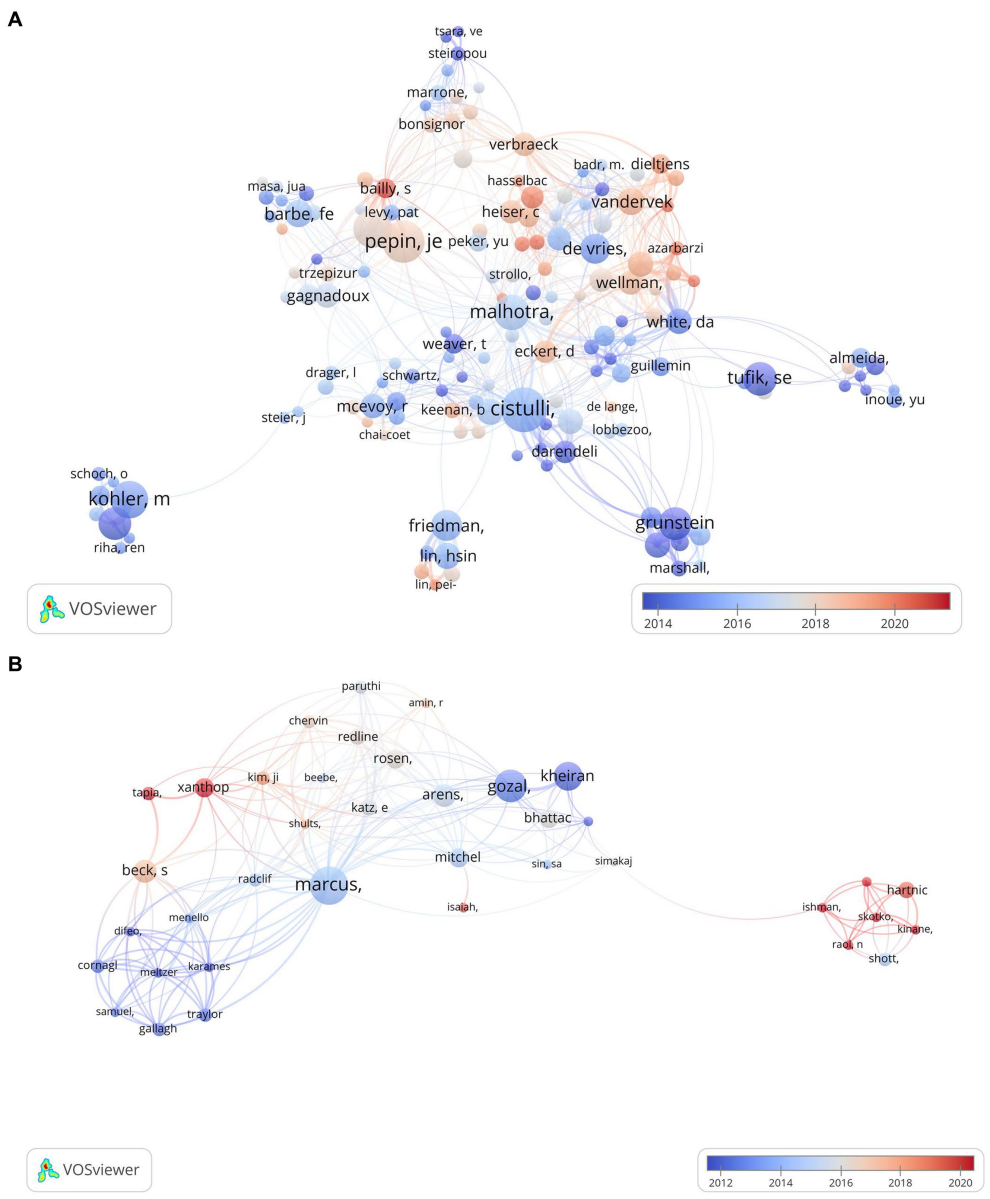
Overlay visualization of authors’ co-authorship of OSA treatment. **(A)** Co-authorship network visualization of authors in adults. **(B)** Co-authorship network visualization of authors in children. Each node represents an author, and the node color represents the temporal distribution (the average year in which the paper was published). The blue color indicates that the author is relatively early in the field, while the red color indicates relatively late research.

After counting the 2,584 articles of adults from a total of 10,867 authors, it was found that Pepin JL published the most papers with 48 articles, and m 5.19 was calculated according to the formula, so there are 237 authors with more than 6 articles identified as the core authors in the field. Table 2 demonstrates the top 10 core authors, their average citations per publication, and the H-index to reflect the authors’ academic contributions. As shown in Figure 4A, Fleury B, Davies RJO, West GH, and 14 other authors produced a series of relevant outputs before 2003, suggesting they have been focusing on the treatment of adult OSA for more than 20 years. Pepin JL, Cistulli PA, Tamisier R, de Vries N, and Malhotra A started to focus on this area after 2013. Malhotra A, Tamisier R, and Pepin JL, who respectively, published their first articles in 2016, 2017, and 2018, are high-quality authors in recent years. Barbe F had the highest average citation per publication (115.79), and Stradling JR had the highest H-index (26). The total number of publications by core authors was 1,647 (63.7%), which was higher than the criterion, indicating that the core authorship of adult OSA treatment research has been initially formed.

**Table 2:** Top 10 core authors of OSA treatment in adults and children.

**Rank**	**Author (Adults)**	**Count**	**Average Citation/Publication**	***H* Index**	**Author (Children)**	**Count**	**Average Citation/Publication**	***H* Index**
1	Pepin JL	48	23.67	20	Marcus CL	15	129	13
2	Cistulli PA	44	57.82	24	Villa MP	12	50	11
3	Stradling JR	37	92.49	26	Gozal D	11	166	11
4	Tamisier R	34	20.5	15	Guilleminault C	10	34.1	7
5	Kohler M	32	42.94	19	Hsu WC	9	15.67	5
6	De Vries N	30	54.71	16	Kang KT	9	15.67	5
7	Grunstein RR	30	85.57	17	Kheirandish-Gozal L	9	118.22	9
8	Malhotra A	30	32.57	16	Lee PL	9	15.67	5
9	Barbe F	28	115.79	19	Weng WC	9	15.67	5
10	Tufik S	28	27.5	18	Arens R	8	168.25	8

A total of 290 articles on pediatric OSA treatment came from 1,259 authors, and we found that Marcus CL published the most papers with 12, and m = 2.6 according to the formula, so the 79 authors with more than three publications were oriented to be the core authors in the field. Figure 4B illustrates that many authors prefer to collaborate with a relatively stable author group. Marcus CL has been focusing on this field since 2014, with the third highest average citation per publication (129) and the highest H-index (13), and has been working closely with core authors such as Gozal D, Arens R, etc., as shown in Table 2. The total number of publications by core authors is 171 (59%), indicating that the core authors of pediatric OSA treatment research have also been initially formed.

### 3.4. Analysis of co-cited references

The co-citation analysis of the adult OSA treatment literature identified a total of 430 publications. Table 3 presents the top 10 most co-cited references. The most frequently cited reference was an observational study by Marin JM, published in *LANCET* in 2005, which investigated the effects of continuous positive airway pressure (CPAP) therapy on long-term cardiovascular outcomes in patients with OSA (38). Another notable reference was a randomized controlled trial published in *NEW ENGL J MED*, which examined the effect of CPAP on cardiovascular outcomes but reported no significant benefits (39). Additionally, three clinical trials focused on CPAP compliance, the efficacy of CPAP compared to oral appliances (OA), and the efficacy of upper airway stimulation (40–42). Peppard PE and Benjafield AV conducted studies assessing the prevalence trends and global burden of OSA (5, 43). Among the identified references, three guidelines were found: two regarding the use of OA and one regarding diagnostic tests for OSA (44–46).

**Table 3:** Top 10 cited references of OSA treatment in adults.

**Rank**	**Count**	**Centrality**	**Author, Year**	**Title**	**Journal**
1	89	0.39	Marin JM, 2005	Long-term cardiovascular outcomes in men with obstructive sleep apnea-hypopnea with or without treatment with continuous positive airway pressure: an observational study	LANCET (IF = 202.731, 2022)
2	88	0.19	Peppard PE, 2013	Increased Prevalence of Sleep-Disordered Breathing in Adults	AM J EPIDEMIOL (IF = 5.363, 2022)
3	79	0.03	McEvoy RD, 2016	CPAP for Prevention of Cardiovascular Events in Obstructive Sleep Apnea	NEW ENGL J MED (IF = 176.079, 2022)
4	72	0.34	Weaver Terri E, 2008	Adherence to Continuous Positive Airway Pressure Therapy: the challenge to effective treatment	ANN AM THORAC SOC (IF = 8.785, 2022)
5	67	0.02	Benjafield AV, 2019	Estimation of the global prevalence and burden of obstructive sleep apnoea: a literature-based analysis	LANCET RESP MED (IF = 102.642, 2022)
6	55	0.12	Ramar K, 2015	Clinical Practice Guideline for the Treatment of Obstructive Sleep Apnea and Snoring with Oral Appliance Therapy: An Update for 2015	J CLIN SLEEP MED (IF = 4.324, 2022)
7	52	0.02	Kapur VK, 2017	Clinical Practice Guideline for Diagnostic Testing for Adult Obstructive Sleep Apnea: An American Academy of Sleep Medicine Clinical Practice Guideline	J CLIN SLEEP MED (IF = 4.324, 2022)
8	50	0.08	Phillips CL, 2013	Health Outcomes of Continuous Positive Airway Pressure versus Oral Appliance Treatment for Obstructive Sleep Apnea	AM J RESP CRIT CARE (IF = 30.528, 2022)
9	49	0.23	Kushida CA, 2006	Practice Parameters for the Treatment of Snoring and Obstructive Sleep Apnea with Oral Appliances: An Update for 2005	SLEEP (IF = 6.313, 2022)
10	48	0.13	Strollo PJ, 2014	Upper-Airway Stimulation for Obstructive Sleep Apnea	NEW ENGL J MED (IF = 176.079, 2022)

The co-citation analysis of pediatric OSA treatment papers yielded a total of 485 publications, and the top 10 most co-cited references are listed in Table 4, with a total of 2 randomized controlled trials (47, 48), 1 multicenter study (49), 1 prospective longitudinal study (50), 3 traditional reviews (51–53) and 3 systematic reviews & meta-analyses (54–56). The major research areas including the impact of adenoid tonsillectomy on treatment outcomes, the diagnosis and treatment of OSA, and efficacy studies of montelukast and myofunctional therapy. The most cited reference is a randomized controlled trial of the efficacy of adenoid tonsillectomy for OSA in children, published by Marcus CL in *NEW ENGL J MED* in 2013 (48).

**Table 4:** Top 10 cited references of OSA treatment in children.

**Rank**	**Count**	**Centrality**	**Author, Year**	**Title**	**Journal**
1	40	0.03	Marcus CL, 2013	A Randomized Trial of Adenotonsillectomy for Childhood Sleep Apnea	NEW ENGL J MED (IF = 176.079, 2022)
2	29	0.10	Marcus CL, 2012	Diagnosis and Management of Childhood Obstructive Sleep Apnea Syndrome	PEDIATRICS (IF = 9.703, 2022)
3	22	0.40	Bhattacharjee R, 2010	Adenotonsillectomy Outcomes in Treatment of Obstructive Sleep Apnea in Children	AM J RESP CRIT CARE (IF = 30.528, 2022)
4	18	0.07	Berry RB, 2012	Rules for Scoring Respiratory Events in Sleep: Update of the 2007 AASM Manual for the Scoring of Sleep and Associated Events	J CLIN SLEEP MED (IF = 4.324, 2022)
5	15	0.11	Friedman M, 2009	Updated systematic review of tonsillectomy and adenoidectomy for treatment of pediatric obstructive sleep apnea/hypopnea syndrome	OTOLARYNG HEAD NECK (IF = 176.079, 2022)
6	13	0.05	Huang YS, 2014	Treatment Outcomes of Adenotonsillectomy for Children with Obstructive Sleep Apnea: A Prospective Longitudinal Study	SLEEP (IF = 6.313, 2022)
7	12	0.04	Lee CH, 2016	Polysomnographic findings after adenotonsillectomy for obstructive sleep apnoea in obese and non-obese children: a systematic review and meta-analysis	CLIN OTOLARYNGOL (IF = 2.729, 2022)
8	11	0.12	Goldbart AD, 2012	Montelukast for Children With Obstructive Sleep Apnea: A Double-blind, Placebo-Controlled Study	PEDIATRICS (IF = 9.703, 2022)
9	11	0.18	Kaditis AG, 2016	Obstructive sleep disordered breathing in 2–18 year-old children: diagnosis and management	EUR RESPIR J (IF = 33.795, 2022)
10	10	0.04	Camacho M, 2015	Myofunctional Therapy to Treat Obstructive Sleep Apnea: A Systematic Review and Meta-analysis	SLEEP (IF = 6.313, 2022)

### 3.5. Analysis of keywords

The top 15 keywords, ranked by frequency and centrality, are presented in Tables 5, 6. For the treatment of OSA in adults, the most prominent keywords include positive airway pressure, CPAP, oral appliances (OA), surgery, and positional therapy. In addition to these, treatments for OSA in children include myofunctional therapy, rapid maxillary expansion, and hypoglossal nerve stimulation.

**Table 5:** Top 15 keywords of OSA treatment in adults by frequency and centrality.

**Rank**	**Count**	**Centrality**	**Keyword**	**Count**	**Centrality**	**Keyword**
1	844	0.02	Positive Airway Pressure	244	0.12	Hypertension
2	775	0.03	OSA	135	0.12	Management
3	661	0.07	CPAP	122	0.11	Mortality
4	477	0.08	Oral Appliance	272	0.1	Daytime Sleepiness
5	316	0.05	Sleep Apnea	266	0.1	Adults
6	273	0.08	Hypopnea	200	0.1	Heart Health
7	272	0.1	Daytime Sleepiness	86	0.1	Disorder
8	266	0.1	Adults	133	0.09	Sleep Disordered Breathing
9	255	0.04	Adherence	93	0.09	Device
10	246	0.07	Cardiovascular Disease	75	0.09	Severity
11	244	0.12	Hypertension	477	0.08	Oral Appliance
12	236	0.08	Blood Pressure	273	0.08	Hypopnea
13	216	0.06	Surgery	236	0.08	Blood Pressure
14	200	0.1	Heart Health	94	0.08	Follow Up
15	196	0.06	Quality of Life	64	0.08	Positional Therapy

**Table 6:** Top 15 keywords of OSA treatment in children by frequency and centrality.

**Rank**	**Count**	**Centrality**	**Keyword**	**Count**	**Centrality**	**Keyword**
1	177	1.44	Obstructive Sleep Apnea Syndrome	177	1.44	Obstructive Sleep Apnea Syndrome
2	21	0.16	Sleep Disordered Breathing	19	0.27	Sleep Apnea
3	19	0.27	Sleep Apnea	21	0.16	Sleep Disordered Breathing
4	15	0.11	Down Syndrome	15	0.11	Down Syndrome
5	14	0.06	Continuous Positive Airway Pressure	13	0.08	Oral Appliance
6	13	0.08	Oral Appliance	14	0.06	Continuous Positive Airway Pressure
7	9	0.01	Positive Airway Pressure	7	0.05	Rapid Maxillary Expansion
8	8	0.02	Adenotonsillar Hypertrophy	3	0.05	Hypoglossal Nerve Stimulation
9	7	0.03	Myofunctional Therapy	2	0.05	Mcgill Oximetry Score
10	7	0.05	Rapid Maxillary Expansion	6	0.04	Apnea Hypopnea Index
11	7	0	Quality of Life	7	0.03	Myofunctional Therapy
12	6	0.04	Apnea Hypopnea Index	8	0.02	Adenotonsillar Hypertrophy
13	6	0.01	Noninvasive Ventilation	4	0.02	Sleep Disorders
14	5	0	Blood Pressure	3	0.02	Post Operative Complications
15	4	0.02	Sleep Disorders	2	0.02	Sleep Study

**Figure 5:**
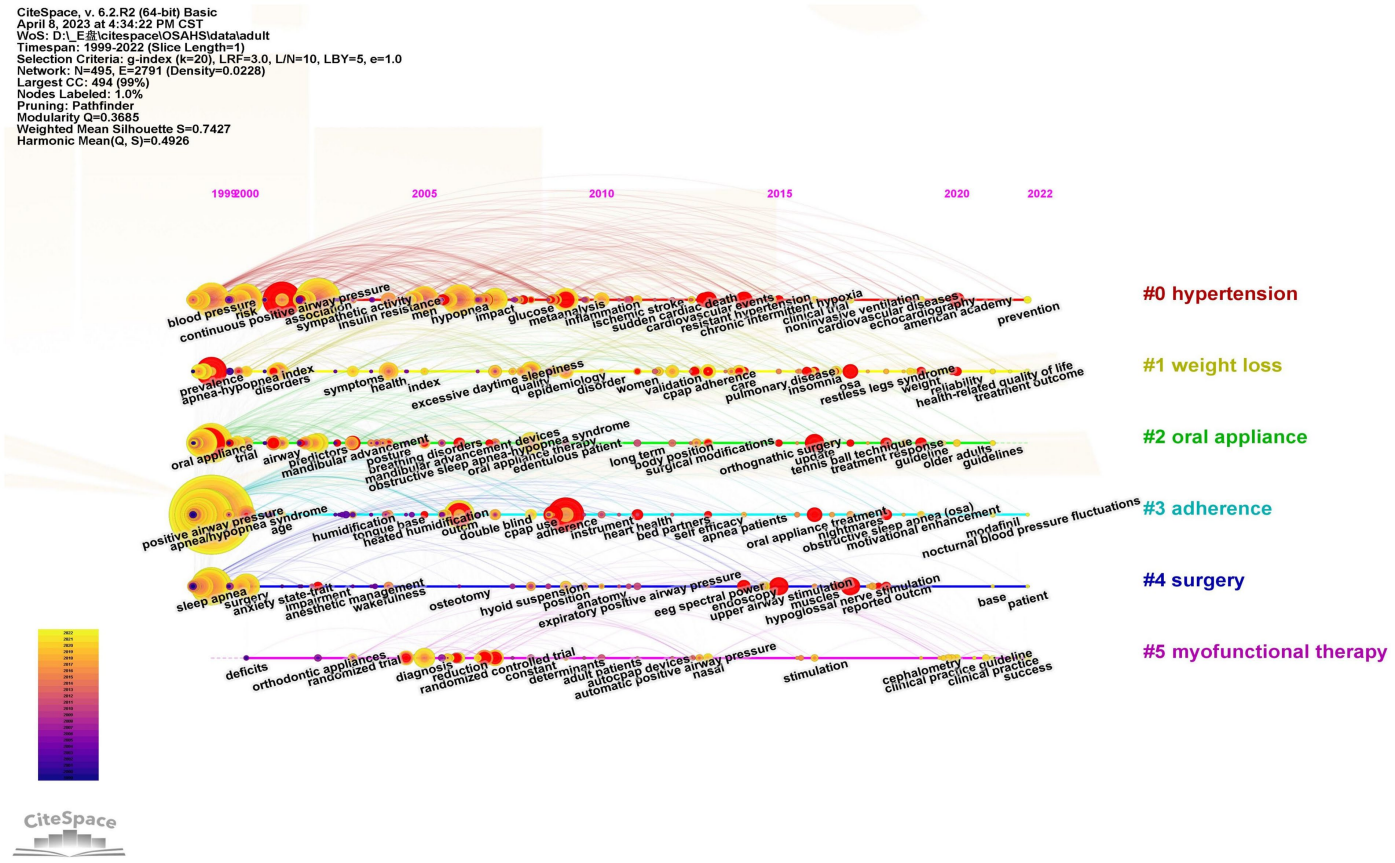
Timeline chart of keywords for OSA treatment in adults. The vertical coordinate is the cluster to which the node belongs, and the horizontal coordinate is the publication time. The size of the node is proportional to the frequency of occurrence of the corresponding keyword, and the color indicates different time periods.

Keywords timeline charts offer improved visualization of the dynamics of research. CiteSpace provides modular values (Q-values) and mean contour values (S-values) to ensure that the mapping is valid (57). The Q-values obtained from the adult and pediatric OSA treatment studies were 0.3685 > 0.3 and 0.6398 > 0.3, respectively, indicating good clustering. The S-values were 0.7427 > 0.7 and 0.9275 > 0.7, respectively, indicating a high degree of homogeneity among the clusters for a reasonable clustering result (26). The clustering of adult OSA treatment studies is shown in Figure 5, with cluster #5 appearing in 2000 and the remaining five clusters appearing in 1999. Cluster #0, labeling “hypertension” is mainly concerned with the relationship and mechanism of OSA comorbidities and complications, including keywords “blood pressure,” “association” “hypertension,” “disease,” “cardiovascular disease,” “hypertension” and so on. Cluster #1, labeling “weight loss” focuses on the prevalence, symptoms, signs, and evaluation indicators of OSA, including “prevalence,” “population,” “disorders “, “obesity,” “quality,” “depression,” etc. Cluster #2, labeling “oral appliance,” cluster #4, labeling “surgery” and cluster #5, labeling “myofunctional therapy” mainly deal with the progression of different treatments from “trail” or “randomized trial “to “clinical practice” or “guidelines.” Cluster #3, labeling “adherence” focuses on studies of adherence factors that influence “positive airway pressure” or “cpap use,” including “age “, “tongue base,” “heated humidification,” “bed partners,” “self-efficacy,” “motivational enhancement,” etc.

**Figure 6:**
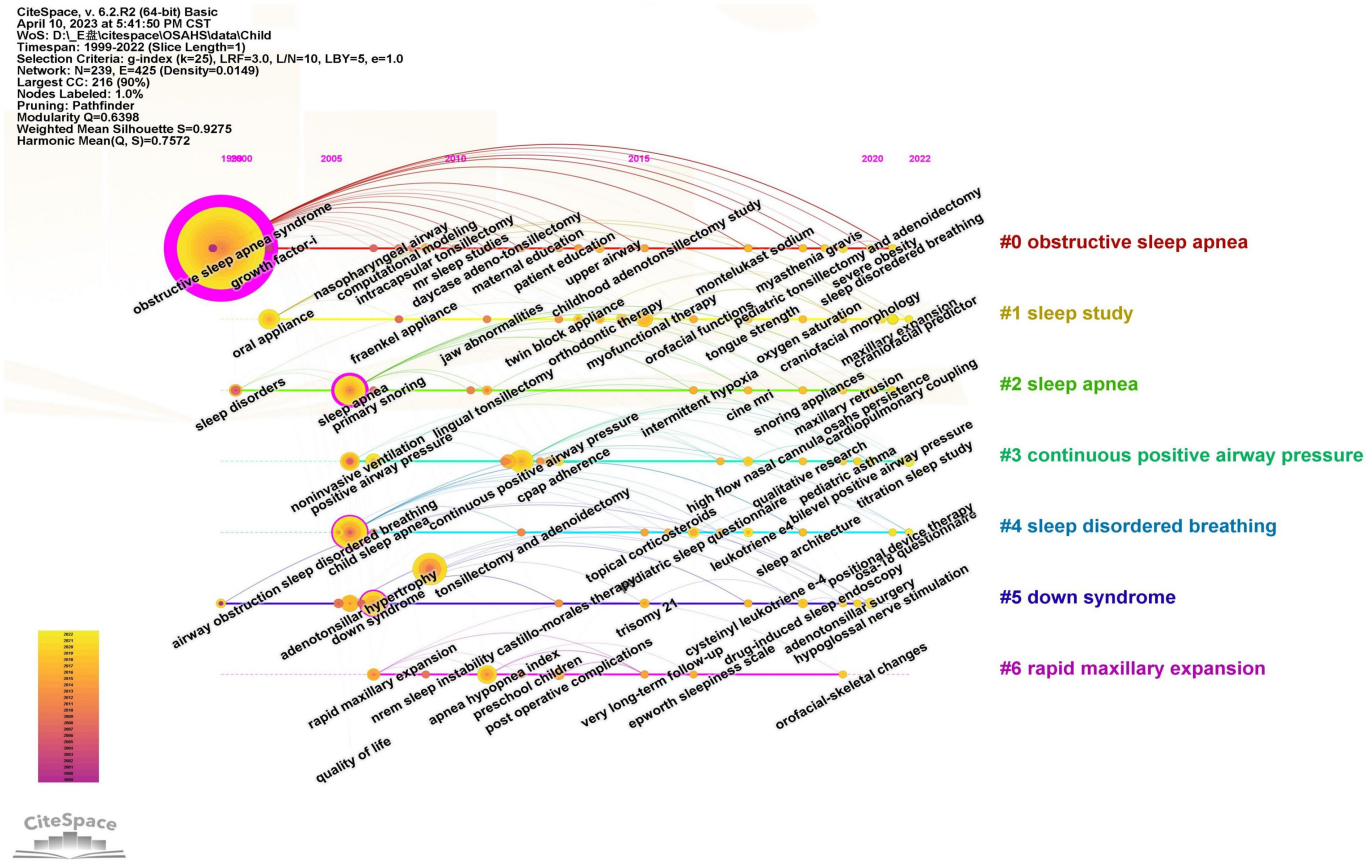
Timeline chart of keywords for OSA treatment in children. The vertical coordinate is the cluster to which the node belongs, and the horizontal coordinate is the publication time. The size of the node is proportional to the frequency of occurrence of the corresponding keyword, and the color indicates different time periods.

The clustering of pediatric OSA treatment studies is shown in Figure 6 with cluster #0, cluster #1, cluster #2, and cluster #5 appearing in 1999–2000 and the remaining three clusters appearing after 2006. Cluster #0 labeling “obstructive sleep apnea” examines the etiology and treatment of OSA at an anatomical level, including keywords such as “nasopharyngeal airway,” “intracapsular tonsillectomy,” “upper airway,” “robotic surgery,” and so on. Cluster #1, labeling “sleep study” and cluster #6, labeling “rapid maxillary expansion” are relevant mainly involving the changes in the structure and function of the orofacial region in patients with OSA and the corresponding therapeutic methods including the keywords “oral appliance,” “myofunctional therapy,” “orthopedic therapy,” etc. Cluster #2, labeling “sleep apnea” primarily involves research on surgical treatments for the major clinical symptoms of OSA including keywords such as “lingual tonsillectomy,” “midline posterior glossectomy,” “bone anchored maxillary protraction,” “intermittent hypoxia” etc. Cluster #3 labeling “continuous positive airway pressure,” is mainly concerned with positive airway pressure ventilation and treatment adherence studies including “continuous positive airway pressure,” “positive airway pressure,” “auto-titrating cpap,” “bilevel positive airway pressure,” “treatment adherence,” “cpap adherence,” etc. Cluster #4, labeling “sleep disordered breathing” is mainly a study of OSA evaluation indicators and methods including keywords like “pediatric sleep questionnaire,” “c-reactive protein,” “osa-18 questionnaire,” and many others. Cluster #5, labeling “down syndrome” mainly focuses on the clinical manifestations and therapeutic method research of the coexistence of genetic diseases and OSA including keywords like “adenotonsillar hypertrophy,” “hypoglossal nerve stimulation,” “adenotonsillar surgery,” “upper airway obstruction,” and “Prader-Willi syndrome.”

**Figure 7:**
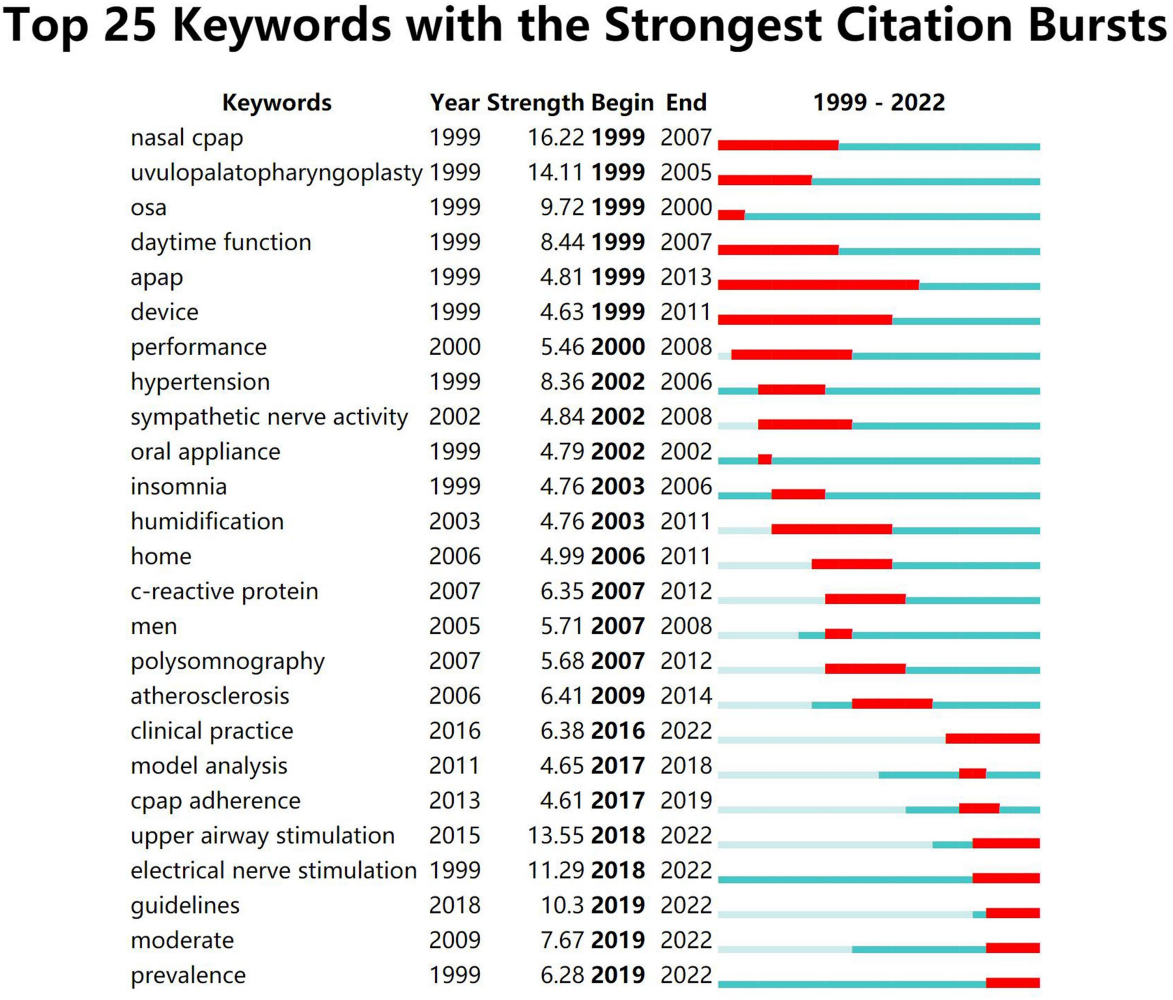
Top 25 burst keywords of OSA treatment in adults. “Begin” represents the beginning year of the keyword in the study timeframe, “End” represents the end year of the keyword, “Strength” represents the intensity of occurrence, the green block represents the unit-year time slice and the red block represents the period of occurrences.

**Figure 8:**
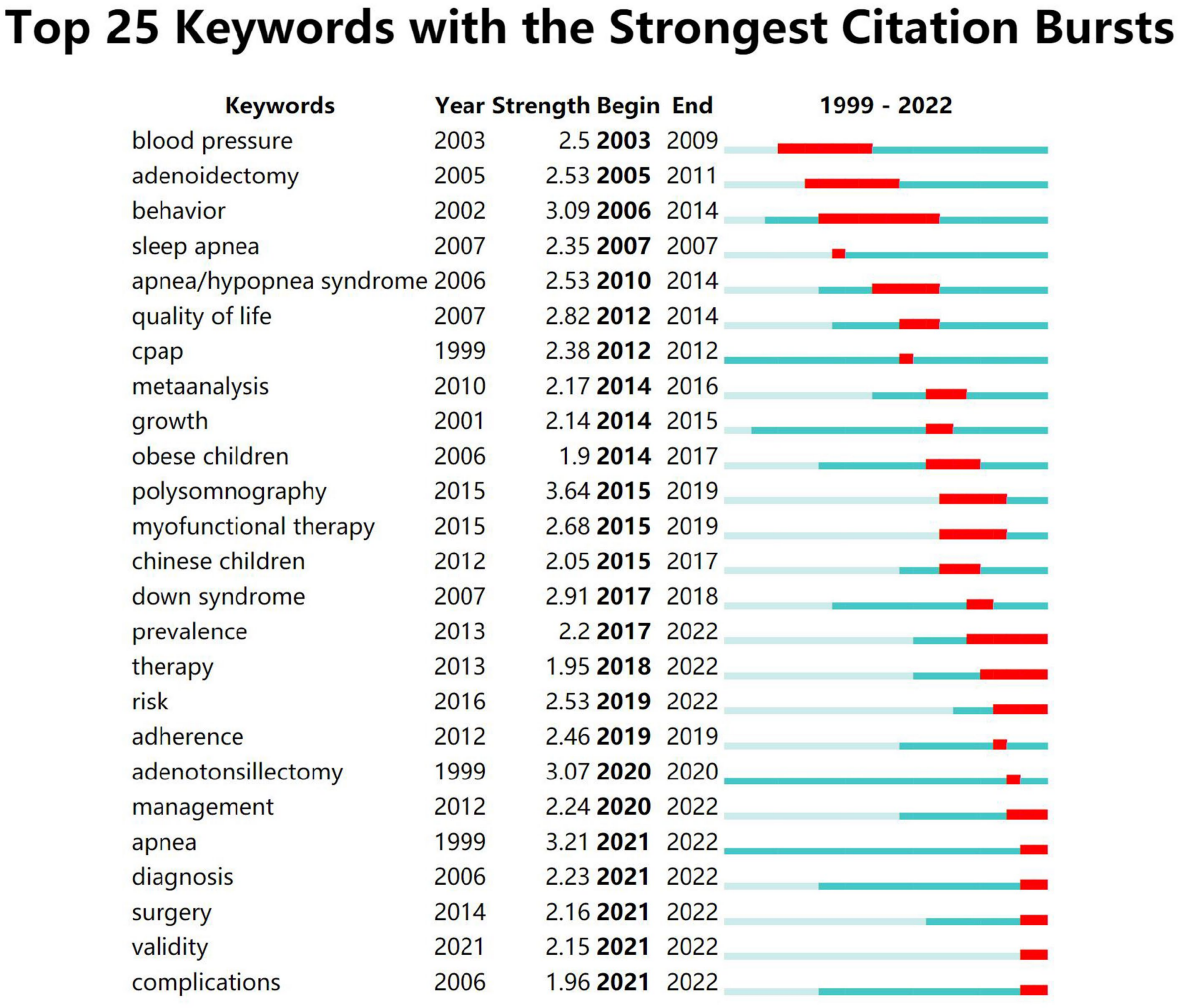
Top 25 burst keywords of OSA treatment in children. “Begin” represents the beginning year of the keyword in the study timeframe, “End” represents the end year of the keyword, “Strength” represents the intensity of occurrence, the green block represents the unit-year time slice and the red block represents the period of occurrence.

As shown in Figure 7, the keywords that emerged with high intensity in the studies for adults are “nasal cpap” (16.22), “uvulopalatopharyngoplasty” (14.11), “upper airway stimulation” (13.55) and “electrical nerve stimulation” (11.29). As shown in Figure 8, the high-intensity keywords that emerged from the research on OSA treatment in children were “polysomnography” (3.64) “apnea” (3.21) “behavior” (3.09) and “adenotonsillectomy” (3.07). In terms of the time span of outbreak duration, outbreaks in adult OSA treatment studies lasted relatively long: “auto positive airway pressure (apap)” -15 years, “device” -13 years, “nasal cpap” -9 years, “uvulopalatopharyngoplasty” -7 years and “electrical nerve stimulation” -5 years, suggesting that they are important therapeutic approaches that have long been of interest to scholars. The explosion of research in the treatment of OSA in children has also continued for a longer period of time, “behavior” -9 years, “adenoidectomy” -7 years and “myofunctional therapy” -5 years, indicating that the above therapies are receiving more attention. According to the temporal ranking, it can be found that the frontier keywords have been changing with the passage of time and have shown a stage-by-stage evolution. The development of research in the field of adult OSA treatment can be roughly divided into three phases: the initial start of research in the 1990s, with treatments based on “nasal cpap” “uvulopalatopharyngoplasty” “apap” and “device,” focusing on the daytime function and performance of patients; then, starting in 2002, “oral appliance” therapy set off a brief research boom, focusing more on the diagnosis of the disease, combined treatment with comorbidities, and the prevention of complications, and began the study of the treatment mechanism of inflammation and neurological aspects; Since 2016, “upper airway stimulation” and “electrical nerve stimulation” therapy have attracted increasing attention from the academic community, and the content of the research is mainly based on clinical practice, modeling, guidelines, and prevalence. There is greater continuity in the development of research in the treatment of OSA in children, with treatments evolving from “behavior” and “adenoidectomy” to “adenotonsillectomy,” “cpap,” “myofunctional therapy” and “suction.” Early diagnosis, complications, surgical treatment, management, epidemiology, and risks are the future research trends.

## 4. Discussion

The study explored the frontier hotspots and evolutionary patterns in OSA therapy in both spatial and temporal dimensions, utilizing a thorough bibliometric analysis using the CiteSpace and VOSviewer tools. The WoS Core Collection database was searched for OSA therapy publications spanning from 1999 to 2022. The analysis revealed a rapid growth in publications related to OSA therapy over the past 3 years, although the total number of publications in pediatric therapy is relatively lower. In adult OSA treatment research, *Sleep and Breathing* emerged as the journal with the highest number of publications. The University of Sydney was identified as the most productive institution, while Pepin JL was ranked as the most productive author. Among the scholars in this field, Barbe F gained recognition as the most cited author. For child-related research, the *International Journal of Pediatric Otorhinolaryngology* had the highest publication count. The University of Pennsylvania and Marcus CL were identified as the most prolific institutions and authors, respectively. Arens R was noted as the most cited author in this domain. Notably, the study highlighted the important countries with high influence and a focus on international collaboration in OSA research, including the USA, China, Australia, Germany, and Italy.

Through co-occurrence analysis of keywords in adult OSA treatment, several commonly used keywords were identified, including positive airway pressure, CPAP, oral appliances (OA), surgery, and positional therapy. CPAP therapy is considered the first-line treatment for most patients with OSA. One of the most influential references in the field is an observational study conducted over more than 10 years, published by Marin JM in 2005. This study demonstrated that CPAP therapy significantly reduces the increased risk of fatal and nonfatal cardiovascular events in severely affected male patients (38). However, it is worth noting that approximately 50% of patients with sleep-disordered breathing who receive CPAP treatment are partially (less than 4 h per night) or completely non-compliant with the therapy’s efficacy (58). Adherence to CPAP is generally moderate, especially among patients with mild OSA (59). As an alternative treatment for patients who cannot tolerate CPAP therapy, OA or mandibular advancement devices are commonly used (60). Nonetheless, there remains considerable uncertainty regarding the exact mechanism of action of OA on the underlying physiology. Studies have demonstrated that OA significantly improves passive anatomy, collapsibility, and ventilation during maximal activation of the pharyngeal muscles. However, they also show that non-anatomical features remain unchanged (61). Postural obstructive sleep apnea refers to a subgroup of OSA in which there are more respiratory events while sleeping in the supine position compared to the non-supine position. Postural therapy, which aims to reduce the time spent in the supine position during sleep, has been shown to effectively decrease the AHI (apnea-hypopnea index) in this subgroup (62). However, there is a lack of long-term efficacy studies on this treatment modality (63).

Through the co-occurrence analysis of keywords in pediatric OSA treatment, several hot keywords were identified, including CPAP, OA, surgery, myofunctional therapy, rapid maxillary expansion, and hypoglossal nerve stimulation. Among the factors contributing to anatomical stenosis, adenotonsillar hypertrophy was found to be the most common cause of OSA in children (64). As a result, adenotonsillectomy has become the first-line treatment option (65). One of the most frequently cited articles in this domain is a randomized controlled trial published by Marcus CL in 2013, which investigated the efficacy of adenoid tonsillectomy for OSA in children (48). Oral appliances have also been considered as a therapeutic option for children, although further study is needed to establish precise indications and follow-up protocols due to craniofacial growth and development (66).

Keyword clustering and emergence analysis have identified new trends in OSA treatment for both adults and children. Firstly, orofacial myofunctional therapy has emerged as a promising approach. Originally originating from speech function training, this therapy aims to improve the function and performance of the upper airway through repetitive isotonic and isometric exercises. By increasing the strength and tone of the upper airway muscles and preventing collapse during sleep, orofacial myofunctional therapy improves the signs and symptoms of OSA patients (54, 67, 68). This therapy exhibits high adherence rates and has been shown to improve CPAP adherence when combined with CPAP treatment (69). It is often recommended as an important alternative treatment or adjunctive to improve the efficacy of moderate to severe OSA (70), including maintaining long-term postoperative outcomes (71). Future studies should consider using standardized exercises to explore the pathophysiology and mechanism of action of orofacial myofunctional therapy for OSA. Secondly, hypoglossal nerve stimulation has gained attention as a promising and safe treatment approach. This method involves stimulating the hypoglossal nerve during inspiration to activate the upper airway dilator muscles and prevent obstruction during sleep (72, 73). Hypoglossal nerve stimulation can be used as an alternative to CPAP for the treatment of moderate-to-severe OSA (74). It has been shown to significantly reduce AHI and the scores of Epworth sleepiness scale in adults (42), and its long-term efficacy and adherence are favorable. Although most published data on hypoglossal nerve stimulation for OSA has focused on children with Down syndrome, these studies have demonstrated improvements in AHI and quality of life as measured by OSA-9, with moderate effect sizes (75, 76). Future studies should aim to extend these results to children without Down syndrome, including younger children in the study population. Lastly, cognitive and behavioral changes have been recognized as essential for OSA treatment due to its chronic nature. Adherence to treatment is crucial, regardless of the treatment modality. Adopting new health behaviors, such as physical activity routines or medication regimens, can be challenging and influenced by social, emotional, and cognitive factors (77). Enhancing patient education through telephone support or home visits has been shown to effectively improve CPAP adherence compared to standard care (78). Motivational interviews with postoperative OSA patients have also demonstrated significant advantages in changing patients’ lifestyles, promoting weight control, and improving long-term postoperative outcomes compared to routine postoperative health education (79).

With the increasing prevalence of obesity in the population, the management of OSA has emerged as a significant public health concern, which includes a higher utilization of healthcare services and a heavier public health burden due to unintentional injuries (80, 81). Clinicians have also paid more attention to the potential for multi-organ and systemic damage. Through visual mining, this study has examined previous prominent and emerging therapeutic approaches for OSA, highlighting the treatment challenges posed by its systemic and heterogeneous nature. For clinicians, a personalized approach to apply, based on a pathophysiological perspective, is crucial for disease management and prognosis. Healthcare administrators can provide high-quality, step-wise, and cost-effective healthcare to OSA patients by establishing multidisciplinary teams and implementing a hierarchical decentralization of healthcare resources.

## 5. Limitations of the study

Using bibliometric methods and analyzing articles published from 1999 to 2022, this study aimed to identify global trends and research hotspots in the field of OSA treatment, providing insights for future scientific research. However, it is important to note several limitations of this study. First, the study relied on the WoS Core Collection database and did not include other databases. Consequently, there may be relevant papers and information that were not captured in the analysis, potentially introducing some degree of selection bias. Secondly, the study only considered publications in the English language, which may further contribute to language and publication bias. Lastly, while the study provided a macroscopic overview of the field by examining countries, journals, institutions, authors, and keywords, it might have missed some important, detailed aspects of OSA treatment. Considering these limitations, future studies should aim to incorporate a broader range of databases, include non-English publications, and delve into more detailed aspects of OSA treatment to provide a comprehensive and accurate understanding of the field.

## 6. Conclusion

This study uses a combination of CiteSpace and VOSviewer software to visualize the activity and collaboration of countries, institutions, and authors in the field of OSA treatment, as well as hot topics and frontier new perspectives. Over the past two decades, the depth and breadth of research in the field of OSA treatment have expanded rapidly. CPAP and surgery are currently the main methods for the treatment of OSA in adults and children. In terms of development trends, since OSA is a multi-heterogeneous disease with complex etiology and pathophysiological variations, the measurement of phenotypic characteristics will enable clinicians to come closer to individualized treatments for patients with OSA. In the future, multidisciplinary combined targeted therapy will be the focus and difficulty of research.

## Data availability statement

The original contributions presented in the study are included in the article/supplementary material, further inquiries can be directed to the corresponding authors.

## Ethics statement

Ethical review and approval was not required for the study on human participants in accordance with the local legislation and institutional requirements. Written informed consent from the participants was not required to participate in this study in accordance with the national legislation and the institutional requirements.

## Author contributions

XY: Conceptualization, Data curation, Visualization, Writing – original draft. YW: Methodology, Conceptualization, Writing – review & editing. SX: Conceptualization, Data curation, Writing – original draft, Writing – review & editing. JC: Data curation, Visualization, Writing – original draft. YL: Data curation, Visualization, Writing – original draft. JZ: Methodology, Writing – review & editing.
